# Sudden swelling and redness of the toe

**DOI:** 10.1002/ccr3.1115

**Published:** 2017-09-21

**Authors:** Vera Tengattini, Michela Magnano, Annalisa Patrizi, Iria Neri

**Affiliations:** ^1^ Department of Experimental, Diagnostic and Specialty Medicine Division of Dermatology University of Bologna Bologna Italy

**Keywords:** Edema, erythema, fingers, ischemia, toes

## Abstract

The presence of history of redness and swelling of toe in an infant without history of trauma is typical of hair threat tourniquet syndrome. The treatment simply involves incision and removal of hair fibers. Physicians should be aware of this syndrome because early diagnosis and treatment avoid serious complications.

An 8‐month‐old girl with a 2‐week history of increasing swelling of her right fourth toe (Fig. 1) associated with irritability. The clinical modification had appeared suddenly, and no foot trauma was reported. The X‐ray was normal.

**Figure 1:**
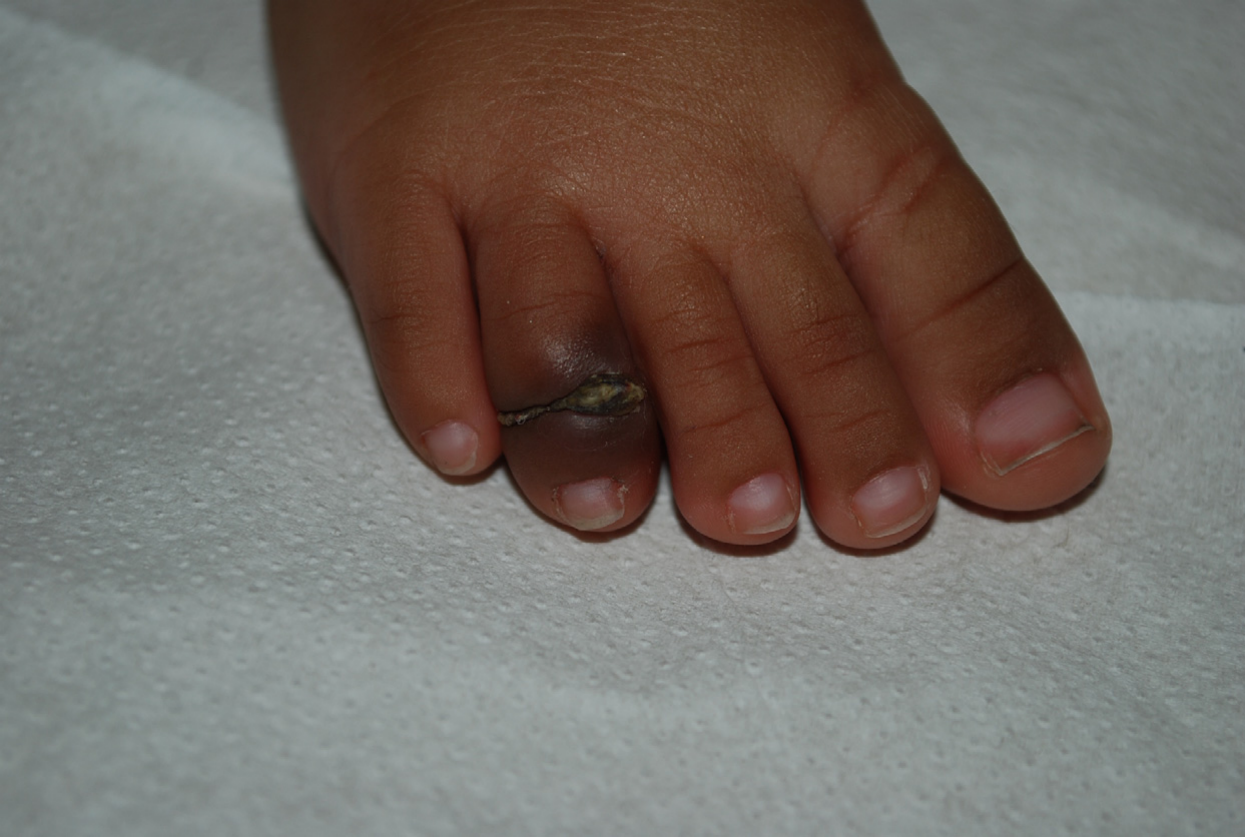
Physical examination revealed a linear crust lesion on the dorsum of the right fourth toe with strangulation of the distal part of the toe, characterized by the presence of edema and erythema.

What would you do next?

- Remove the crust lesion
- Oral amoxicillin/clavulanic acid
- Topical fusidic acid
- Program a biopsy

## Discussion and Outcomes

**Figure 2:**
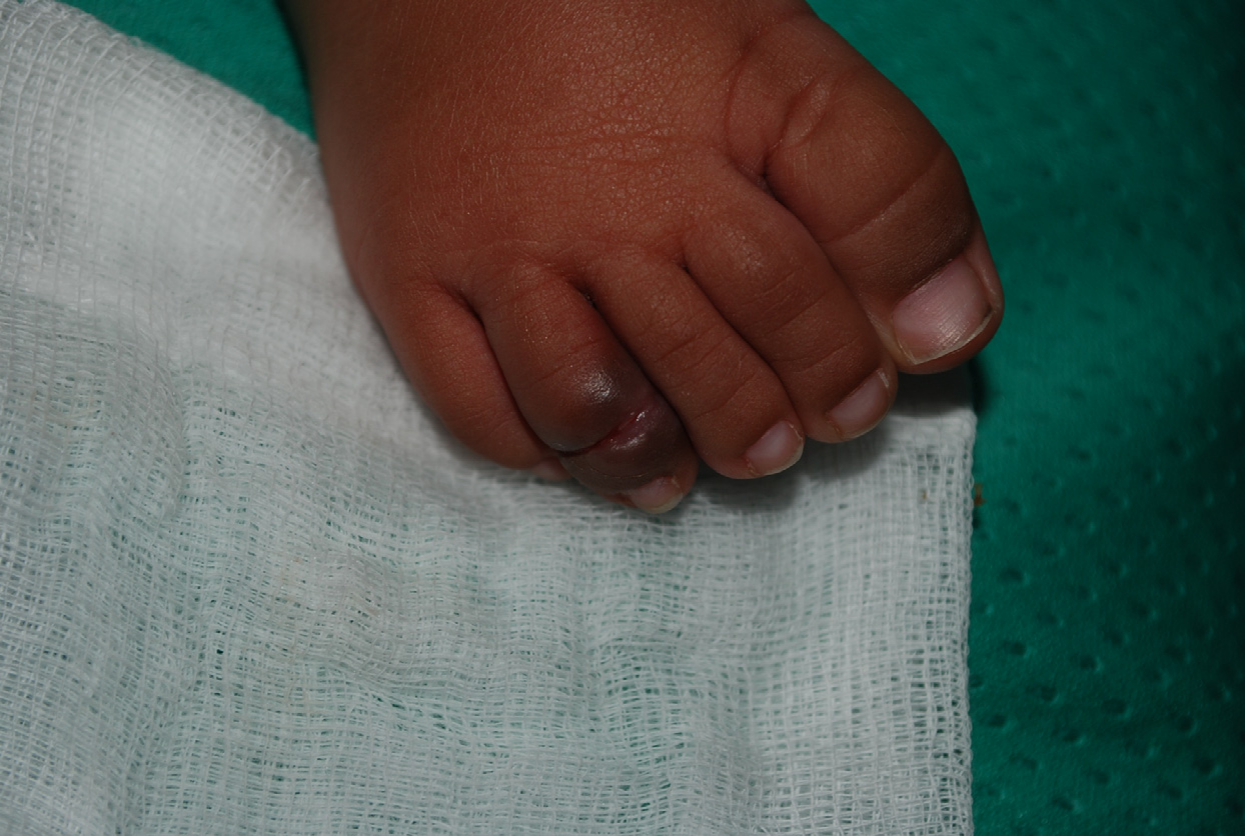
The hair coil, after removal.

**Figure 3:**
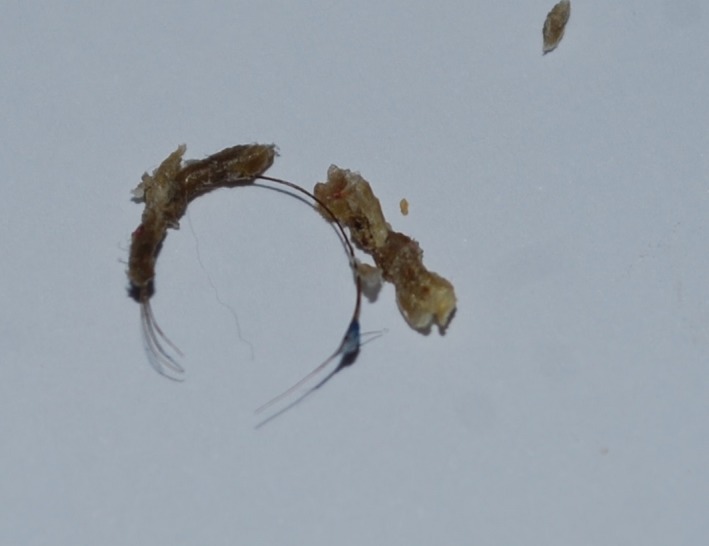
Physical examination after crust removal.

The key clinical feature of this case is the presence of history of redness and swelling of toe in an infant without history of trauma. Hair threat tourniquet syndrome is a clinical condition first described by Quinn in 1971 1. It is rare and potentially severe acquired surgical emergency usually observed in children due to hair 2. The circumferential strangulation impairs lymphatic and venous drainage causing edema, arterial occlusion till ischemy, necrosis, and finally autoamputation. In our case, the crust was gently removed (Fig. 2) and revealed the hair coil (Fig. 3).

Hyperemia and edema regressed after 1 day.

## Authorship

VT, MM, AP, IN: all authors, have participated in the work; (1) substantial contributions to conception and design, acquisition of data, or analysis and interpretation of data; (2) drafting the article or revising it critically for important intellectual content; and (3) final approval of the version to be published.

## Conflict of Interest

The authors have no conflict of interest to disclose.
